# An audit of radiation doses received by paediatric patients undergoing computed tomography investigations at academic hospitals in South Africa

**DOI:** 10.4102/sajr.v24i1.1823

**Published:** 2020-10-16

**Authors:** Cornelis M. van der Merwe, Nasreen Mahomed

**Affiliations:** 1 Department of Radiology, Faculty of Health Sciences, University of the Witwatersrand, Johannesburg, South Africa

**Keywords:** Radiation dose, Computed tomography (CT), Paediatric patients, Diagnostic Reference Level (DRL), Computed Tomography Dose Index_volume_ (CTDI_volume_)

## Abstract

**Background:**

Diagnostic reference levels (DRLs) are a crucial element of auditing radiation doses in paediatric computed tomography (CT). Currently, there are no national paediatric CT DRLs in South Africa.

**Objectives:**

The aim of this article was to establish local paediatric DRLs for CT examinations at two academic hospitals and to compare paediatric CT radiation output levels with established DRLs in the developed and developing world.

**Method:**

Computed Tomography Dose Index_volume_ (CTDI_vol_) and dose length product (DLP) values were collected from CT examinations performed at two university hospitals for patients aged 0–15 years, during 01 November 2016–30 April 2017. The 75th percentile of the data distribution was calculated for each CT examination type and age group, further categorised into routine working hours and after-hours for both hospitals and statistically compared.

**Results:**

Of the 1031 CT examinations performed, CT brain examination was the most common (755/1031; 72.23%). DLP values were increased in the after-hours categories compared to regular working hours at both hospitals. The largest increase was in the 0–1 year age group (150.56%). With the exception of CT Chest and CT abdomen in the 0–1 year age group, the CTDI_vol_ and DLP values compared favourably to international standards.

**Conclusion:**

Most of the calculated DRLs are acceptable and internationally comparable. This likely indicates effective reduction techniques and protocols. Computed tomography body examination protocols for 0–1 year patients should be reviewed. Strategies should be implemented to limit higher doses in after-hours examinations.

## Introduction

Since its advent in 1971, computed tomography (CT) has added tremendous value for diagnosis and establishing treatment plans for patients. Since then, there has been an exponential increase in the usage of CT.^1^ This increase is because of several factors, including, but not limited to, rapid evolvement of technology and advancements in hardware and software, which led to improved image quality and reduced duration for CT examinations.^2,3^ In addition, the geopolitical and socio-economic trends since the late 1990s also contributed to greater access to medical resources and equipment, specifically in the industrialised world.^4^ The number of CT scanners per million people in Japan increased from 14.36 in 1980 to 107.14 in 2017. This increase has been the most significant in the developed world; however, an increase in the amount of CT scanners was also observable in the developing world, for example, in Turkey, where the number of CT scanners increased from 4.89 per million people in 2002 to 14.53 per million people in 2016.^4^ The advances in availability and increase in applications also made CT investigations popular in the paediatric patient population. In the Netherlands, the total number of paediatric CT scan examinations increased from 7731 in 1990 to 26 023 in 2012.^5^ Similar trends were established in the rest of the developed world.^6^

Even though there was suspicion about harmful effects of ionising radiation on the human body shortly after Roentgen took his first radiograph in 1895, the first International Radiation Congress only discussed possible radiation protection standards in 1925. In the aftermath of the Second World War, the International Commission on Radiation Protection (ICRP) and the United Nations Scientific Committee on the Effects of Atomic Radiation were formed and have since then played a major role in radiation research and protection.^7^ The concept of keeping radiation dose ‘as low as reasonably achievable’ (ALARA) has been around since 1915 and is compatible with the medical ethical mantra of ‘first do no harm’.^8^ Furthermore, evidence from the Second World War and radiation incidents has proved that the younger the patient is, the higher is the risk for adverse radiation effects. The increased risk is because of the presence of more undifferentiated cells, and the cells have a higher mitotic rate as well as a longer mitotic future.^1,9^

The ICRP has recommended diagnostic reference levels (DRLs) for all diagnostic and interventional radiological procedures since 1991 as a measure to ensure radiation protection.^6^ The Image Gently Alliance and campaign, which started in 2007, promoted the ALARA principle and since then has become one of the primary considerations in paediatric imaging.^3^ Since 2007, there has been a reduction in the annual increase of paediatric CT examinations in the developed world.^5^ This reduction is likely because of the successes of radiation awareness programmes.

Following recommendations by the ICRP to establish DRLs, there have been a significant number of audits and DRL proposals in the developed world.^10^ As of 2013, the European Diagnostic Reference Levels for Paediatric Imaging (PiDRL) workshop has driven a campaign to establish European DRLs.^10^

There have been very few studies or audits on paediatric CT doses to establish CT-specific DRLs in the developing world. There is only one study from South Africa auditing CT doses in a tertiary hospital on non-contrasted paediatric brain CT scans.^11^

South Africa is considered the most industrialised country and the second largest economy in Africa but has one of the highest levels of inequality. Most of the population is medically underserviced because of resource constraints in the public health sector.^12^ Apart from a heavy workload required from the radiological equipment, there are also restraints on human resources and quality control. In addition, the absence of established DRLs limits the ability to do routine audits to ensure optimal radiation protection. It is therefore of utmost importance to audit paediatric CT doses in South Africa to establish DRLs.

The aim of this study was to establish local paediatric DRLs for CT examinations in two major academic hospitals affiliated to the University of the Witwatersrand, Johannesburg, South Africa.

Other objectives were to audit radiation doses and compare paediatric CT investigations’ radiation output levels to established DRLs in the developed and developing world. An additional objective was to evaluate whether there was any difference between the hospitals, as well as between regular work hours and after-hours.

## Materials and methods

### Design

The study was designed as a retrospective, descriptive study.

### Dosimetry

European guidelines suggest the usage of the Computed Tomography Dose Index_volume_ (CTDI_vol_) and dose length product (DLP) as CT dose descriptors.^10^ CTDI_100_ is a linear measure of the dose distribution in a 10 cm ionization chamber inserted into a 16 cm phantom for paediatric CT. The weighted CTDI (CTDI_w_) is calculated by establishing the CTDI_100_ for the centre and the periphery of a cylinder and combining these. In helical CT scanning, the dose is inversely related to the pitch (number of rotations of the gantry per distance moved by the examination bed). Computed Tomography Dose Index_w_ divided by the pitch equals CTDI_vol_, which is expressed in the international system of units (SI units) as milligray (mGy). Dose length product is the product of the length of the scanned area with the CTDI_vol_^13^ and is expressed in milligray-centimetre (mGy*cm).

### Study population

The data collected were the CTDI_vol_ and DLP values for each CT examination in paediatric patients (age less than 15 years) during the 6-month period from 01 November 2016 to 30 April 2017 at the following hospitals: Charlotte Maxeke Johannesburg Academic Hospital (CMJAH) and Rahima Moosa Mother and Child Hospital (RMMCH). These hospitals are situated within the City of Johannesburg Municipality in Gauteng province, South Africa.

### Data collection

Data were retrieved from the Picture Archiving and Communication System (PACS) from CMJAH and the local area network at RMMCH. Data were categorised and tabled for each CT scanner and further categorised according to the type of study and the age group. The CT brain data were also categorised into three different time categories as follows: weekdays (Monday to Friday from 08:00 to 16:00), after-hours (00:00–08:00 and 16:00–24:00 from Monday to Friday) and weekends and public holidays (00:00–24:00 on Saturday and Sunday, as well as on public holidays).

The categories were chosen as per recommendations made by the European Diagnostic Reference Levels for Paediatric Imaging Workshop in 2013.^10^ All CT investigations were included and categorised according to the anatomical region of interest. The data could not be categorised according to indication, as the indication was not available on the database. The age groups were divided into 0 to < 1 year; 1 year to < 5 years, 5 years to < 10 years and 10 years to < 15 years. The European Commission suggests categorising the CT body examinations according to weight, but patients’ weight was not available from the database. The time of day and day of the week was recorded for each study.

### Data analysis

The distribution of the CT examinations in this study sample was calculated using frequencies.

For statistical analysis of the CTDI_vol_ and DLP values, only the single-phase CT examinations were included in the study sample. Radiation output data for CT orbits, CT paranasal sinuses, CT musculoskeletal, CT whole spine and CT peripheral angiography were excluded from further analysis as the sample sizes were too small for statistical significance.

Data sets were categorised for CT brain, CT temporal bones, CT neck, CT cervical spine, CT chest, CT trunk and CT abdomen. The dose distribution for CT brain in each age category was determined for each hospital. Data for each of the other CT examination types were combined for the two hospitals.

The mean, average, median and 75th percentile of the data distribution, with confidence intervals (CIs), were then calculated for each category for each examination type. Local DRLs are defined as the 75th percentile of the data distribution.^10^ The local DRLs in this study were the 75th percentile value for each category, rounded up to the nearest single digit for CTDI and the nearest 5 for DLP.

The data were then compared to similar studies with local and national DRLs.

In addition, the CT brain results were compared between the two hospitals according to the difference in CTDI_vol_ and DLP 75th percentile values between the two hospitals for each age group and time category, using the Fisher’s exact test. Furthermore, the quantile regression method was used to compare the 75th percentiles in different groups by calculating the 95% CI for the difference between percentiles. If the 95% CI for the difference did not contain 0, the percentiles were significantly different.

### Ethical considerations

Ethical approval to conduct the study was obtained from the Human Research Ethics Committee (Medical) of the University of the Witswatersrand (approval number: M170634).

## Results

### Distribution and frequency of computed tomography examinations

The audit period for the 6 months from 01 November 2016 to 30 April 2017 included 1031 paediatric CT examinations from RMMCH and CMJAH.

Computed tomography brain examinations (755/1031; 73.23%) were the most common CT examination, followed by CT of the abdomen, which amounted to 82/1031 (7.95%) (see Table 1).

**TABLE 1:** Total number of scans included in the study per computed tomography examination type for each age category (*n* = 1031).

Examination type	0–1 years	1–5 years	5–10 years	10–15 years	Total
CT Brain	195	263	159	138	755(73.23%)
CT Temporal Bones	1	1	4	10	16(1.55%)
CT Paranasal Sinuses	1	1	2	2	6(0.58%)
CT Orbits	0	0	4	0	4(0.39%)
CT Neck	3	10	8	6	27(2.62%)
CT Cervical Spine	3	17	13	9	43(4.17%)
CT Whole spine	1	2	1	0	4(0.39%)
CT Trunk	5	12	11	8	36(3.49%)
CT Chest	18	10	9	8	45(4.36%)
CT Abdomen	8	24	17	33	82(7.95%)
CT Limbs	0	1	3	5	9(0.87%)
Peripheral CT angiography	3	0	0	2	5(0.48%)

**Total**	**238 (23.08%)**	**341 (33.07%)**	**231 (22.41%)**	**221 (21.44%)**	**1031 (100%)**

CT, computed tomography.

### Radiation doses

From all the study types conducted during the study period, there were only seven study types with enough cases in different age groups to allow data distribution calculation. These included CT brain, CT temporal bones, CT neck, CT cervical spine, CT trunk, CT chest and CT abdomen (total number for analysis = 905). The 75th percentile of both the CTDI_vol_ and DLP of each of these examination types in the various age groups is demonstrated in Table 2, with a 95% CI.

**TABLE 2:** 75th percentile of Computed Tomography Dose Index_vol_ and Dose Length Product for each computed tomography examination type, in each age group, as well as total number of contributing studies per category (*n* = 905).

Study	Age	CTDI_vol_: 75th percentile†		DLP: 75th percentile‡		Nunber of studies
		95%	CI	95%	CI	
CT Brain	0–1 years	20.3	19.68–20.91	311.30	291.94–330.66	169
	1–5 years	20.3	19.72–20.89	362.40	342.38–382.42	238
	5–10 years	22.33	18.18–25.14	457.20	395.78–518.62	156
	10–15 years	32.14	32.10–32.18	746.10	719.41–772.80	124
CT Temporal Bone	5–10 years	41.78	11.31–64.02	305.80	135.73–475.87	4
	10–15 years	57.37	38.19–76.55	547.20	445.24–649.16	10
CT Cervical Spine	0–1 years	13.16	0.92–25.40	303.90	−4.64–612.44	3
	1–5 years	6.44	−4.12–17.00	186.00	−67.56–439.56	17
	5–10 years	7.07	5.53–8.61	186.7	129.87–243.53	13
	10–15 years	8.5	7.42–9.58	227.9	137.53–318.27	9
CT Neck	0–1 years	7.85	7.62–8.08	195.40	59.33–331.47	3
	1–5 years	6.93	−5.43–19.29	215.20	69.09–361.30	10
	5–10 years	7.85	−1.12–16.82	142.30	60.20–224.40	7
	10–15 years	15.99	−1.70–33.68	269.70	166.02–373.38	6
CT Trunk	0–1 years	19.29	13.78–24.8	1362.30	194.15–2530.45	5
	1–5 years	4.73	4.73–4.74	212.7	193.99–231.41	12
	5–10 years	6.51	2.68–10.34	238.80	−58.11–535.71	11
	10–15 years	4.73	1.57–7.89	290.20	178.94–401.46	8
CT Chest	0–1 years	5.66	1.89–9.43	153.50	20.16–286.84	12
	1–5 years	3.27	2.39–4.15	105.10	56.56–153.64	9
	5–10 years	4.73	−3.49–12.95	136.40	85.90–186.90	8
	10–15 years	6.97	3.69–10.25	325.10	205.96–444.24	7
CT Abdomen	0–1 years	6.17	3.52–8.82	191.50	67.36–315.64	4
	1–5 years	4.73	3.52–5.94	187.80	144.28–231.32	18
	5–10 years	4.73	3.21–6.25	203.30	145.18–261.42	14
	10–15 years	8.40	2.64–14.16	371.10	195.10–547.10	26

CTDI_vol_, Computed Tomography Dose Index_volume_; CT, computed tomography; DLP, Dose Length Product.

† , CTDI_vol_ values presented in mGy;

‡ , DLP values presented in mGy*cm.

The CT brain data sets were used to compare the two different hospitals, as well as to evaluate for potential variation in different time categories. At CMJAH, there was an increase in the 75th percentile of the data distribution in the weekend and after-hours group compared to regular weekdays. The greatest increase in dose was in the 0–1-year after-hours group with a 150.56% (691.3 mGy*cm vs. 275.9 mGy*cm) increase in DLP compared to the 0–1-year group during routine weekdays. The second most significant increase in dosage was in the 5–10-year weekend group. Here, there was a 78.07% (760.7 mGy*cm vs. 427.2 mGy*cm) increase in DLP compared to 5–10 years routine weekday group (see Tables 3 and 4).

**TABLE 3:** 75th percentile Computed Tomography Dose Index_vol_ (mGy) of data distribution, categorised for time and age for computed tomography brain examinations at Charlotte Maxeke Johannesburg Academic Hospital (*n* = 515).

Age	Weekdays Regular hours	After-hours	Weekends	Combined
0–1 years	12.91	32.14	12.91	12.91
1–5 years	11.82	12.91	14.33	12.91
5–10 years	19.37	19.37	32.14	19.37
10–15 years	32.14	32.14	32.14	32.14

**TABLE 4:** 75th percentile of Dose Length Product (mGy*cm) data distribution, categorised for time and age for computed tomography brain examinations at Charlotte Maxeke Johannesburg Academic Hospital (*n* = 515).

Age	Weekdays Regular hours	After-hours	Weekends	Combined
0–1 years	275.90	691.30*	304.90	284.10
1–5 years	289.40	329.50	339.35	301.80
5–10 years	427.20	457.20	760.70**	457.20
10–15 years	746.10	834.90	778.60	759.05

* Not statistically significant, as 0 is included in the 95% CI.

** Statistically significant, as 0 is not included in the 95% CI.

Similarly, the data from RMMCH demonstrated an increase in CTDI_vol_ and DRL for after-hours and weekends compared to regular weekdays, although the increase was not as significant as it was at CMJAH. The most pronounced increase in dose was in the 1–5-year after-hours group, with an increase in DLP of 40.46% (570.7 mGy*cm vs. 406.3 mGy*cm). The second highest increase in dose compared to routine weekdays was in the 0–1-year after-hours category, with an increase of 25.92% (418.3 mGy*cm vs. 332.2 mGy*cm) in DLP (see Tables 5 and 6).

**TABLE 5:** 75th percentile Computed Tomography Dose Index_vol_ (mGy) of data distribution, categorised for time and age for computed tomography brain examinations at Rahima Moosa Mother and Child Hospital (*n* = 172).

Age	Weekdays Regular hours	After-hours	Weekends	Combined
0–1 years	20.30	23.00	20.30	20.03
1–5 years	21.65	23.00	21.66	21.65
5–10 years	23.00	23.00	23.00	23.00
10–15 years	35.18	23.00	35.18	35.18

**TABLE 6:** 75th percentile of Dose Length Product (mGy*cm) data distribution, categorised for time and age for computed tomography brain examinations at Rahima Moosa Mother and Child Hospital (*n* = 172).

Age	Weekdays Regular hours	After-hours	Weekends	Combined
0–1 years	332.20	418.30*	359.70	345.00
1–5 years	406.30	570.70**	380.25	410.60
5–10 years	437.70	569.70	497.90	497.90
10–15 years	647.45	450.90	683.00	647.45

* Not statistically significant, as 0 is included in the 95% CI.

** Statistically significant, as 0 is not included in the 95% CI.

**FIGURE 1:**
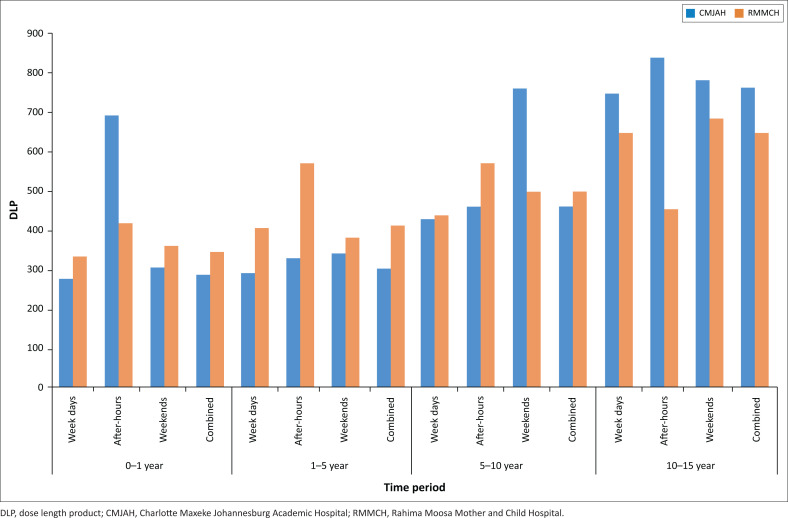
Comparative 75th percentile for dose length product in mGy*cm for each time category for computed tomography brain at Charlotte Maxeke Johannesburg Academic Hospital and Rahima Moosa Mother and Child Hospital (*n* = 687).

The comparison of the dosages during CT brain investigation between the two hospitals revealed in general a lower DLP at a lower DLP at CMJAH for the 0–1y, 1–5y and 5–10y groups, compared to RMMH (statistically significant in the 0–1y and 5–10y groups only, as 0 is not included in the 95% CI). The 10–15-year stratified groups demonstrated lower DLP values at RMMCH compared to CMJAH (see Figure 1).

### Comparison to other studies and diagnostic reference levels

The combined DLP and CTDI_vol_ 75th percentile values were compared to DRLs from the European guidelines, UK, Germany, Japan, Kenya and Brazil^10,14,15,16,17,18^ (see Tables 7 and 8).

**TABLE 7:** Computed Tomography Dose Index_volume_ 75th percentiles (mGy) of Johannesburg hospitals compared to international diagnostic reference levels.

Examination	Johannesburg		EDRL†	UK‡	Germany§	Japan¶	Kenya††	Brazil‡‡
	95%	CI						
**CT Brain**								
0–1 years	20.30	19.69–20.91	24	25	30	38	38	18
1–5 years	20.3	19.72–20.88	28	40	35	47	50	30
5–10 years	21.66	18.18–25.14	40	60	50	60	55	35
10–15 years	32.14	32.10–32.18	50	-	55	-	-	44
**CT Chest**								
0–1 years	5.66	1.90–9.42	1.4–1.8	-	1.7	11	-	5
1–5 years	3.27	2.39–4.15	1.8–2.7	-	2.6	14	11	7
5–10 years	4.73	−3.49–12.95	2.7–3.7	-	4	15	-	-
10–15 years	6.97	3.52–8.82	3.7–5.4	-	6.5	-	11	-
**CT Abdomen**								
0–1 years	6.17	3.51–8.82	3.5	-	-	11	-	4
1–5 years	4.73	3.52–5.94	3.5–5.4	-	-	16	11	5
5–10 years	4.73	3.21–6.25	5.4–7.3	-	5	17	-	-
10–15 years	8.47	2.63–14.16	7.3–13	-	7	-	-	-

EDRL, Europe diagnostic reference levels; CT, computed tomography.

† , European Commision (2018) Radiation Protection No 185.^10^

‡ , Doses from Computed Tomography (CT) Examinations in the UK-2011.^14^

§ , Bundesamt fur Strahlenschutz (2016).^15^

¶ , Japan Network for research and Information on Medical exposures (2015).^16^

†† , National Diagnostic Reference Level Initiative for Computed Tomography examinations in Kenya (2016).^17^

‡‡ , A Contribution to the Establishment of Diagnostic Reference Levels in Computed Tomography in Brazil (2015).^18^

**TABLE 8:** Dose Length Product 75th percentile (mGy*cm) of Johannesburg hospitals compared to international diagnostic reference levels.

Examination	Johannesburg		EDRL†	UK‡	Germany§	Japan¶	Kenya††	Brazil‡‡
	95%	CI						
**Brain**								
0–1 years	311.30	291.94–330.65	300	350	300	50	1005	290
1–5 years	362.40	342.38–382.42	385	650	450	660	1395	550
5–10 years	457.20	395.77–518.62	505	620	650	850	1608	670
10–15 years	746.10	719.41–772.79	650	-	800	-	-	880
**Chest**								
0–1 years	153.50	20.16–286.84	35–50	-	25	210	-	64
1–5 years	105.10	56.56–153.64	50–70	-	55	300	215	130
5–10 years	136.40	85.90–186.90	70–115	-	110	410	-	-
10–15 years	325.10	205.96–444.24	115–200	-	200	-	453	-
**Abdomen**								
0–1 years	191.50	67.36–315.64	45–120	-	-	220	-	110
1–5 years	187.80	144.28–231.32	120–150	-	-	400	764	170
5–10 years	203.30	145.18–261.41	150–210	-	185	530	-	220
10–15 years	371.10	195.10–547.10	210–480	-	310	-	-	-

EDRL, Europe diagnostic reference levels.

† , European Commission (2018) Radiation Protection No 185.^10^

‡ , Doses from Computed Tomography (CT) Examinations in the UK-2011.^14^

§ , Bundesamt fur Strahlenschutz (2016).^15^

¶ , Japan Network for research and Information on Medical exposures (2015).^16^

†† , National Diagnostic Reference Level Initiative for Computed Tomography examinations in Kenya (2016).^17^

‡‡ , A Contribution to the Establishment of Diagnostic Reference Levels in Computed Tomography in Brazil (2015).^18^

The CTDI_vol_ and DLP values for CT brain were found to be less than the comparative DRL values in most cases, except for the DLP values compared to the European DRL in the age category for 10–15 years.

The CTDI_vol_ and DLP values for CT chest were higher than the European DRLs, but better than those from Japan, Kenya and Brazil. The exception was the increased value compared to Brazil in the 0–1-year age category.

The CTDI_vol_ values for CT abdomen were lower than the international DRLs, except for the 0–1-year category, which was higher than the European Diagnostic Reference Levels (EDRL) and Brazilian values. The 0–1-year category for CT abdomen DLP values was also higher than those from Brazil. Although only the 10–15-year category DLP values were within the EDRL range, the rest of the values were lower than that of the other international DRLs.

## Discussion

### Distribution and frequency of computed tomography examinations

The higher number of investigations at the CMJAH compared to RMMCH was expected, as it is considered a central hospital in South Africa, a level 1 trauma centre and major referral centre in the country.^19^ The CT brain percentage of total investigations was marginally higher in comparison to international studies in the developed world, whereas the CT abdomen percentage compared to the CT utilisation trends in other countries was similar.^5,6,20^ The reason for the higher percentage of CT brains performed at the studied facilities is likely because the initial neuroimaging investigation in the public health sector of South Africa for a child presenting with the first episode of convulsion is a CT brain instead of a magnetic resonance imaging (MRI) brain, as recommended by the American Academy of Neurology.^21^ Computed tomography brain for neurological disease in South Africa is a reasonable initial radiological investigation, as the incidence of neurological infections is higher than that of developed countries.^22^ Magnetic resonance imaging availability and anaesthetic support are limited in the South African public health sector. Furthermore, CMJAH is a level 1 trauma centre and will have an increased percentage of CT brains for trauma indications.

The increase in CT of the cervical spine after the age of 1 year is expected in a level 1 trauma centre. The number of temporal bone CT investigations in the 5–10- and 10–15-year age groups is consistent with previous studies, which demonstrated the majority of patients with temporal bone pathology to be between the ages of 11 and 20 years.^23^

### Radiation doses

Both hospitals demonstrated an increase in the DLP values of CT brain during after-hours and some of the weekend categories. During after-hours, there is less staff present on the floor and often fewer senior staff to guide procedures, which could lead to an incorrect choice of parameters or selection of scan area, with a resultant increase in radiation dose to the patient. The increase in values is more significant in CMJAH than in RMMCH. The Radiology Department at RMMCH is almost an exclusive paediatric radiology department, with staff trained in paediatric radiology. On the contrary, the Radiology Department at CMJAH is a large combined adult and paediatric academic radiology department. At CMJAH, there are dedicated time slots for paediatric CT examinations during the week, but after-hours urgent paediatric CTs are performed in between adult patient CTs, which could lead to an incorrect parameter and CT protocol selection when examining children. Previous research has shown that probability exists for a significant DLP variation between radiographers even in the setting of a dedicated paediatric hospital.^24^

Although the finding of increased DLP values in the 0–1-year age group was considered not to be statistically significant, follow-up investigation in this age group is suggested, as the findings might suggest clinical significance.

The increased CTDI_vol_ and DLP values for RMMCH compared to CMJAH could be ascribed to hardware and software variables between the two departments. Charlotte Maxeke Johannesburg Academic Hospital Philips machines have more detector rows (64 and 128) as well as utilisation of the i-Dose software by Philips.^25,26^

### Comparison to other studies and diagnostic reference levels

The CTDI_vol_ and DLP values for CT brain compare well against previously established DRLs and suggest consistent application of well-developed protocols at the different facilities.

Most of the higher CTDI_vol_ and DLP values in the 0–1-year age group for CT abdomen were associated with higher kV settings. The South African Society of Paediatric Imaging suggests the reduction of kV settings in paediatric examinations.^27^ The CT abdomen studies with CTDI_vol_ and DLP values comparable to international DRL ranges were performed with a reduction of kV from 120 to 100. It is suggested that the CT abdomen protocol for both RMMCH and CMJAH should be reviewed, adjusted and applied to all cases.

Although the CTDI_vol_ and DLP values for CT chest compare well against some of the international DRLs, it is also suggested that the protocols for CT chest should be reviewed and adjusted.

A discrepancy in the comparison of CTDI_vol_ and DLP values for a specific examination in a specific age category is likely because of a larger-than-expected pre-selected scan area for the particular study.^24,25^ This and the fact that the CT brain values compared better than the CT chest and abdomen values could be a result of using age, rather than weight, as an input parameter for CT chest and abdomen examination in children.^10^

### Outcome

From the data analysis, DRLs are proposed for the most frequent examinations. Local DRLs are not suggested for 0–1-year age group, except for CT brain, as the 75th percentile values are higher than the older age groups and compare unfavourably to international DRLs (Table 9).

**TABLE 9:** Proposed local diagnostic reference levels for Computed Tomography Dose Index_volume_ and Dose Length Product for paediatric computed tomography examinations.

Study	Age	CTDI_vol_: 75th percentile (mGy)	DLP: 75th percentile(mGy*cm)
CT Brain	0–1 years	21	315
	1–5 years	21	365
	5–10 years	23	460
	10–15 years	33	750
CT Temporal bones	5–10 years	40	315
	10–15 years	56	515
CT Cervical Spine	1–5 years	7	190
	5–10 years	8	190
	10–15 years	9	230
CT Neck	1–5 years	7	200
	5–10 years	7	145
	10–15 years	15	260
CT Trunk	1–5 years	5	215
	5–10 years	6	235
	10–15 years	6	285
CT Chest	1–5 years	4	110
	5–10 years	7	145
	10–15 years	7	290
CT Abdomen	1–5 years	5	185
	5–10 years	5	230
	10–15 years	9	460

CTDI_vol_, Computed Tomography Dose Index_volume_; CT, computed tomography; DLP, Dose Length Product.

**FIGURE 2:**
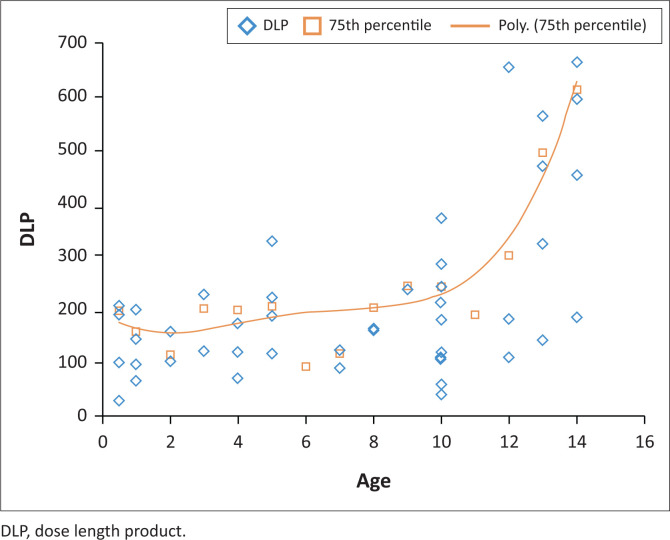
Polynomial exponential curve for the purpose of presenting diagnostic reference levels for computed tomography abdomen for specific ages. The data set used in this graph was the dose length product for computed tomography abdomen examinations, corrected for each year in age (*n* = 82).

**FIGURE 3:**
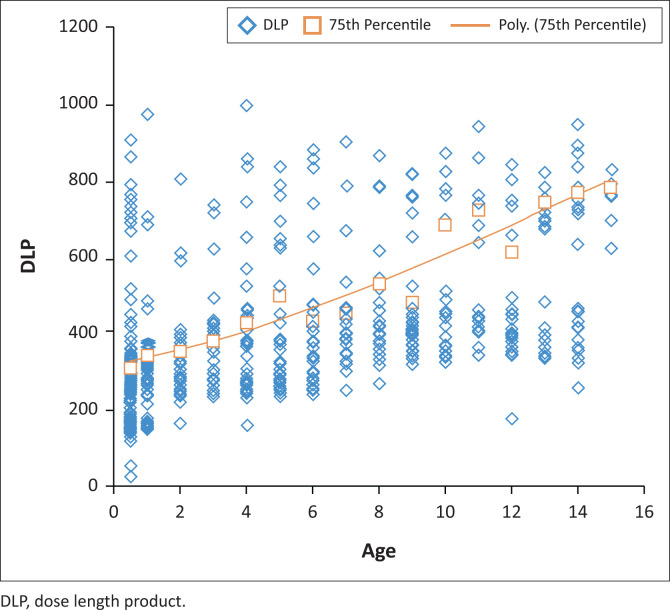
Polynomial exponential curve for the purpose of presenting diagnostic reference levels for computed tomography brain for specific ages. The data set used in this graph was the dose length product for computed tomography brain examinations, corrected for each year in age (*n* = 687).

In recent similar international studies and according to the European guidelines, it is proposed that DRLs could be presented in a graph format instead of tabular format. See the DRL graph and curve for CT abdomen in Figure 2 and for CT brain in Figure 3. This type of graph is created by plotting all the different values for each age on an *x*:*y* scatter plot, establishing the 75th percentile for each age and creating a polynomial, exponential graph.^28^

Presentation of a DRL in a graph format can aid in the comparison of results and also could be an easy visual reference in the department.

### Limitations

One of the limitations of this study is that the European guidelines suggest that body CTs should be categorised according to weight, but the weights were not documented on PACS for RMMCH and CMJAH during the study period. Further limitations included the significant percentage of multi-phasic CT scans, specifically in the 0–1-year age group, which limited the statistical significance of the findings.

## Conclusion

Overall, the CTDI_vol_ and DLP values for the studies are comparable with most of the international DRLs. Computed tomography chest and abdomen protocols should be revised, specifically in the 0–1-year age groups. A suggestion would be to use weight as an input parameter instead of age for CT chest and abdomen examinations.

The DRL values in Table 9 are suggested as local DRLs for the University of the Witwatersrand academically affiliated hospitals as well as their referral hospitals.

The results of this study will be presented to the South African Society of Paediatric Imaging to aid in the establishment of national DRLs for paediatric CT examinations.
